# Radioligand therapy for pediatric patients with solid tumors

**DOI:** 10.1093/bjr/tqag161

**Published:** 2026-07-07

**Authors:** Leonor Teles, Nelleke Tolboom, Alex J Poot, Khalil Ramdhani, Sabine L A Plasschaert, Johannes H M Merks, Max M van Noesel, Arthur J A T Braat

**Affiliations:** Department of Radiology and Nuclear Medicine, Princess Maxima Center for Pediatric Oncology, 3584 CS Utrecht, The Netherlands; Department of Solid Tumors, Princess Maxima Center for Pediatric Oncology, 3584 CS Utrecht, The Netherlands; Department of Radiology and Nuclear Medicine, Princess Maxima Center for Pediatric Oncology, 3584 CS Utrecht, The Netherlands; Department of Radiology and Nuclear Medicine, University Medical Center Utrecht/Wilhelmina Children’s Hospital, 3584 CX Utrecht, The Netherlands; Division Imaging and Oncology, Utrecht University, 3584 CX Utrecht, The Netherlands; Department of Radiology and Nuclear Medicine, Princess Maxima Center for Pediatric Oncology, 3584 CS Utrecht, The Netherlands; Department of Radiology and Nuclear Medicine, University Medical Center Utrecht/Wilhelmina Children’s Hospital, 3584 CX Utrecht, The Netherlands; Division Imaging and Oncology, Utrecht University, 3584 CX Utrecht, The Netherlands; Department of Radiology and Nuclear Medicine, University Medical Center Utrecht/Wilhelmina Children’s Hospital, 3584 CX Utrecht, The Netherlands; Division Imaging and Oncology, Utrecht University, 3584 CX Utrecht, The Netherlands; Department of Neuro-oncology, Princess Maxima Center for Pediatric Oncology, 3584 CS Utrecht, The Netherlands; Department of Solid Tumors, Princess Maxima Center for Pediatric Oncology, 3584 CS Utrecht, The Netherlands; Division Imaging and Oncology, Utrecht University, 3584 CX Utrecht, The Netherlands; Department of Solid Tumors, Princess Maxima Center for Pediatric Oncology, 3584 CS Utrecht, The Netherlands; Division Imaging and Oncology, Utrecht University, 3584 CX Utrecht, The Netherlands; Department of Radiology and Nuclear Medicine, Princess Maxima Center for Pediatric Oncology, 3584 CS Utrecht, The Netherlands; Department of Radiology and Nuclear Medicine, University Medical Center Utrecht/Wilhelmina Children’s Hospital, 3584 CX Utrecht, The Netherlands; Division Imaging and Oncology, Utrecht University, 3584 CX Utrecht, The Netherlands; Department of Nuclear Medicine, Netherlands Cancer Institute, 1066 CXAmsterdam, The Netherlands

**Keywords:** radioligand therapy, radiopharmaceuticals, neuroblastoma, sarcoma, osteosarcoma, brain tumors, theranostics

## Abstract

Despite significant advances in the treatment of childhood cancers, progress in improving outcomes for many pediatric solid tumors remains limited, particularly in patients with relapsed or metastatic disease. Current treatment strategies, including surgery, chemotherapy, and external beam radiotherapy, can achieve disease control but are often associated with substantial acute and long-term toxicities that affect long-term development and impact the overall quality of life of survivors. Radioligand therapy (RLT) has emerged as a promising targeted therapeutic strategy that combines tumor-specific ligands with radioactive isotopes, enabling the selective delivery of radiation to cancer cells while minimizing damage to surrounding healthy tissues. In adult oncology, RLT has demonstrated substantial clinical benefit in several malignancies, although this has not yet translated to pediatric cancers. Early clinical studies have explored the use of RLT in pediatric solid tumors, particularly in neuroblastoma, but the available evidence remains fragmented and is largely limited to small series. Therefore, this review aims to summarize the current clinical evidence on the use of RLT in pediatric solid tumors, including relevant molecular targets, radiopharmaceuticals, clinical outcomes, and safety profiles, and to discuss the key challenges and future directions for the development of RLT in pediatric oncology.

## Introduction

Pediatric solid tumors are a heterogeneous group of malignancies, accounting for approximately 50%-60% of childhood cancers and 70% of cancer-related deaths.^1^^,^^2^ Although survival outcomes in pediatric oncology have improved substantially over the past decades, these gains have been largely restricted to hematological malignancies (eg, leukemia, lymphoma), while progress in the treatment of solid tumors, including CNS tumors, has been considerably slower. Survival rates for pediatric sarcoma have remained largely unchanged over the past decades, despite intensified multimodal treatment strategies.^3^ In addition, solid tumor survivors frequently experience long-term treatment-related complications, including impaired growth, neurocognitive deficits, organ dysfunction, and reduced quality of life.^4^^,^^5^ Taken together, the limited survival improvements and persistent complications demonstrate that current therapies are insufficient. Thus, new therapies that selectively target tumor cells while sparing normal, developing tissues are urgently needed.

One of these therapies is radioligand therapy (RLT), which uses a targeting molecule (ligand) linked to a radionuclide to selectively deliver ionizing radiation, such as alpha or beta particles, directly to tumor cells, while sparing healthy tissues (Figure 1). Beyond the direct cellular damage (eg, DNA strand breaks), some radionuclides also irradiate neighboring tumor cells, which is advantageous to treat heterogeneous tumors. Within a theranostic framework, RLT pairs an imaging radiopharmaceutical (labeled with a positron- or gamma-emitter radionuclide) using a therapeutic one (labeled with an alpha- or beta-emitter radionuclide) on a similar ligand (Figure 2). The imaging radiopharmaceutical identifies eligible patients and guides the subsequent delivery of the therapeutic radiopharmaceutical, ensuring personalized and targeted treatment.^6^

**Figure 1:**
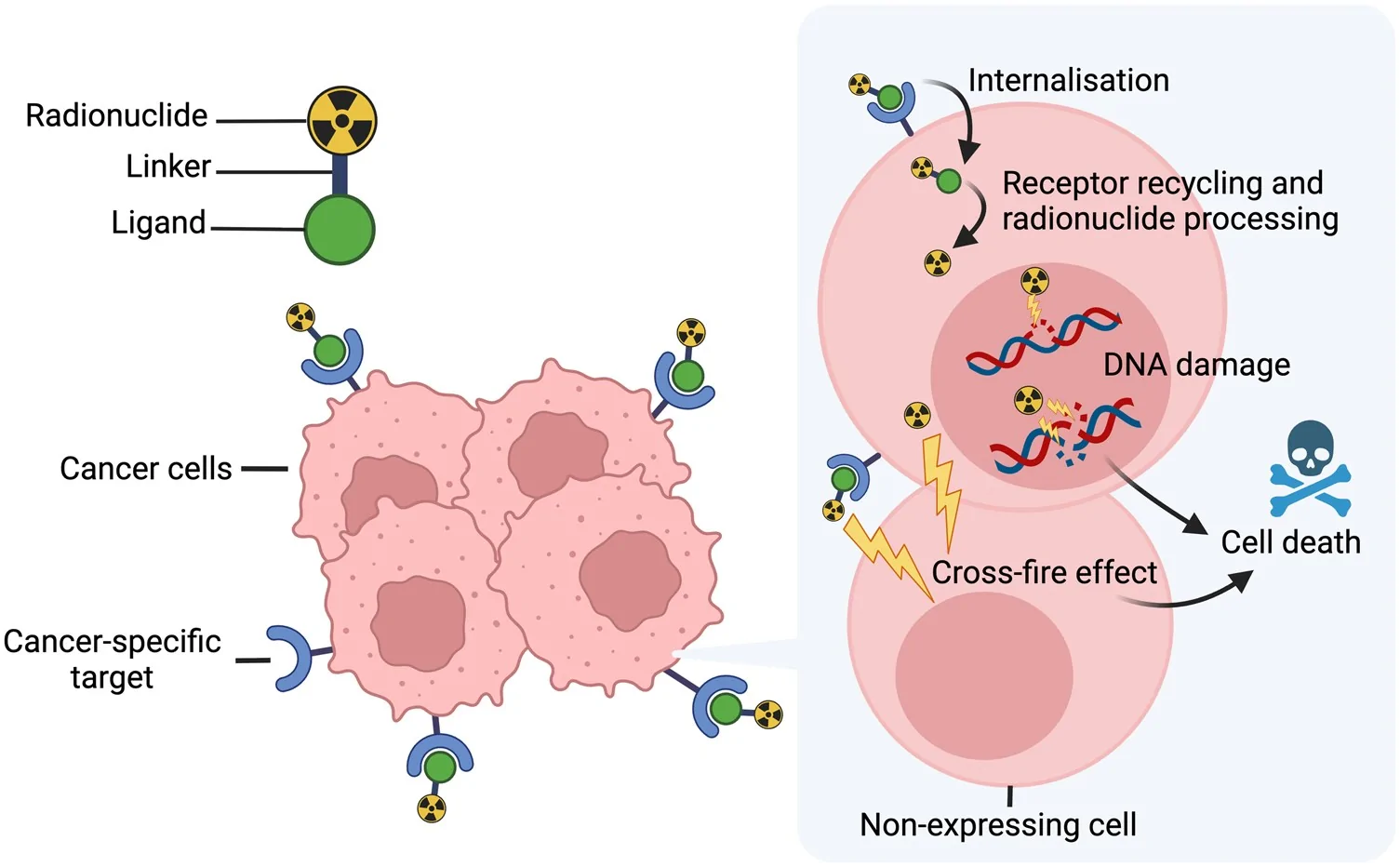
Mechanism of action of radioligand therapy (RLT).

**Figure 2:**
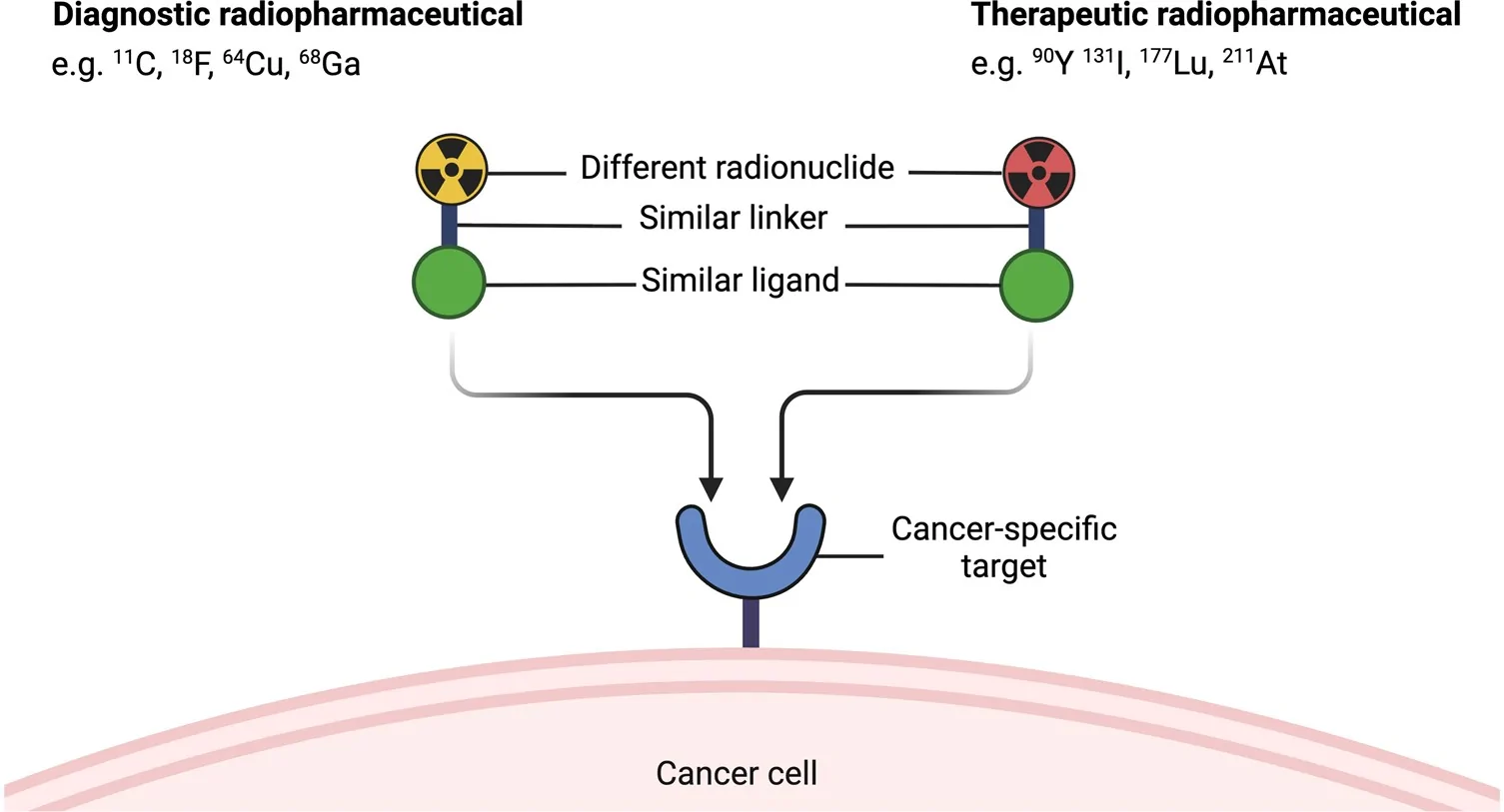
Theranostic framework. The same targeting molecule (ligand) and linker can be used for diagnosis and therapy by switching the radionuclide attached.

RLT and theranostics have fundamentally transformed adult oncology, evolving from the historical use of iodine-131 (^131^I) for the imaging and treatment of thyroid cancer—the birthplace of nuclear medicine—into the modern era of precision targeting. A landmark example is peptide receptor radionuclide therapy (PRRT) with [^177^Lu]Lu-DOTATATE for gastroenteropancreatic neuroendocrine tumors. Although it is an established therapy for advanced somatostatin receptor-positive disease,^7^ phase III data from the NETTER-2 trial have further expanded [^177^Lu]Lu-DOTATATE’s indication to first-line settings for well-differentiated, higher-grade tumors.^8^ In metastatic castration-resistant prostate cancer, PSMA-targeted RLT (eg, [^177^Lu]Lu-PSMA-617) was initially approved for patients who progressed after androgen receptor pathway inhibitors and taxane-based chemotherapy (VISION trial).^9^ Recent phase III data from the PSMAfore trial support approval extension to earlier use (before chemotherapy), based on PSMA tumor expression on imaging.^10^ Transarterial radioembolization (TARE), a form of locoregional RLT using yttrium-90 microspheres, also has a central role in unresectable or chemotherapy-refractory liver disease (eg, colorectal/neuroendocrine metastases) and is considered a first- or second-line therapy for hepatocellular carcinoma.^11^

Despite these advances, the translation of RLT and theranostics from adult to pediatric oncology has been slow, due to the rarity of pediatric tumors, the limited number of validated targets, small patient populations, and concerns over long-term radiation effects on developing organs. Historically, the clinical application of RLT in pediatric solid tumors has been dominated by radioactive iodine as an adjuvant in differentiated thyroid carcinoma and iodine-131-l metaiodobenzylguanidine ([^131^I]mIBG) for the treatment of neuroblastoma.^12^^,^^13^ However, advances in tumor biology are expanding the range of targets, and improvements in radiochemistry are providing novel radioligands for both new and known targets.^14^

In this review, we summarize the clinical evidence regarding RLT in pediatric solid tumors. We analyze the status of biological targets and radiopharmaceuticals, highlight ongoing clinical trials, and discuss future directions for integrating RLT into precision treatment strategies for children with solid tumors, including CNS tumors.

## Methods

Electronic databases such as PubMed, Google Scholar, and Embase, were searched using predefined strings (Supplemental Data). The reference lists of relevant articles were manually screened to identify further eligible studies. Included articles were limited to clinical studies, case series, and case reports published in English, from 2000 to 2026, investigating radiopharmaceuticals used for the treatment of pediatric solid tumors. Information on planned and ongoing clinical trials was obtained from the ClinicalTrials.gov registry. Studies addressing imaging or diagnostic uses were considered outside of the scope.

## Neural crest and neuroendocrine tumors

Neuroblastoma, paraganglioma, and pheochromocytoma are tumors that arise from the embryonic neural crest, a transient cell population that gives rise to the peripheral nervous system and adrenal medulla.^15^ In these tumors, one of the main targets for RLT is the norepinephrine transporter (NET), which mediates the uptake of norepinephrine into the cells. Meta-iodobenzylguanidine (mIBG), a norepinephrine analog, uses the transporter and selectively accumulates in the tumor cells (Figure 3). mIBG is used as a theranostic radiopharmaceutical, targeting tumor cells for imaging, when radiolabeled with iodine-123 (^123^I), and for therapy, when radiolabeled with iodine-131 (^131^I).^16^

**Figure 3:**
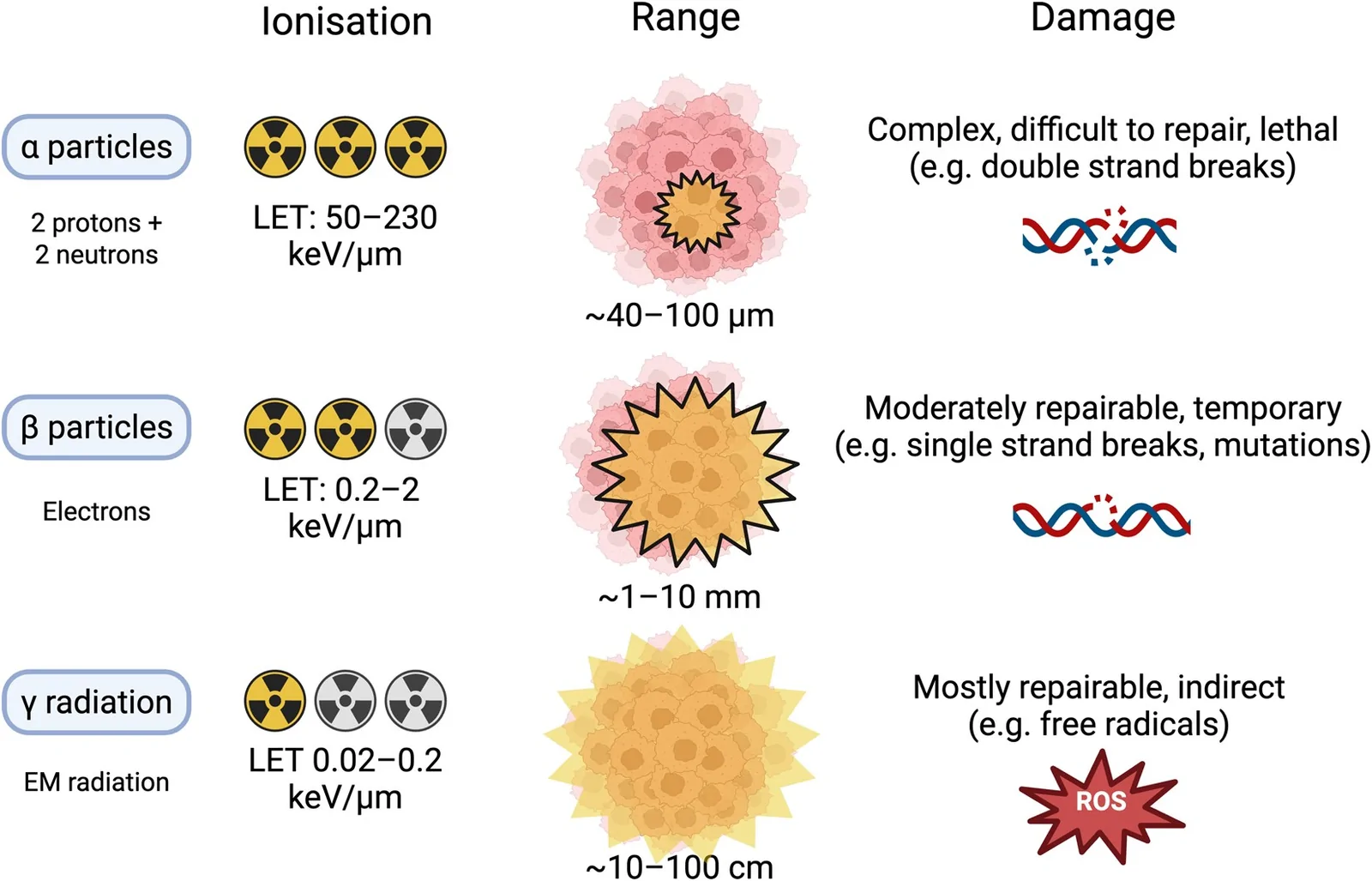
Properties of alpha, beta, and gamma radiation.

In neuroblastoma, [^131^I]mIBG therapy initially focused on the refractory and relapsed setting^17–23^ but has more recently been employed as part of upfront, induction, and consolidation treatments.^24–26^ Although the populations are heterogeneous, previous meta-analyses have demonstrated pooled objective response rates (ORR) of approximately 30%-39%.^27^ Efforts to enhance the therapeutic efficacy of [^131^I]mIBG have included combination with radiosensitizers, immunotherapy, and chemotherapy.^28–33^ A recent example of these combinations is the NANT 2011-01 phase II randomized trial. [^131^I]mIBG monotherapy (arm A) was compared with [^131^I]mIBG with vorinostat (arm B), and [^131^I]mIBG with vincristine and irinotecan (arm C).^34^ Arm B showed the highest response rate (32%) with manageable toxicity. Another example is the French multicenter phase I/II trial MIITOP (NCT00960739), in which the combination of [^131^I]mIBG with topotecan with a personalized dosing schedule achieved an ORR of 13% and an event-free survival (EFS) of 17%, in a heavily pretreated population.^35^ Then, in the NANT 2017-01 (NCT03332667) phase I trial, [^131^I]mIBG was combined with dinutuximab (with or without the addition of vorinostat).^36^ In the absence of vorinostat (arm A), no dose-limiting toxicities (DLTs) occurred, but when vorinostat was added (arm B), one DLT and additional significant toxicities were observed. Although both arms showed the same response (ORR 42%), around one fifth of patients in arm A progressed, while no progression was observed in arm B. Thus, adding vorinostat to the [^131^I]mIBG and dinutuximab combination may improve disease control, but it is also associated with more toxicity. Finally, instead of focusing on the refractory/relapsed setting, a recent pilot study (NCT01175356) showed the feasibility of integrating [^131^I]mIBG at induction combined with myeloablative busulfan/melphalan (BuMel) in newly diagnosed high-risk patients.^37^ This study laid the groundwork for the ongoing randomized trial ANBL1531 (NCT03126916), which will further test the addition of [^131^I]mIBG to induction therapy (Table 1). Other ongoing efforts, such as the phase II OPTIMUM trial and phase I MiNivAN trial, will further evaluate the combination of [^131^I]mIBG with radiosensitizers and immunotherapy, respectively.

**Table 1:** Ongoing radioligand therapy trials in patients with neuroblastoma and other neural crest/neuroendocrine tumors.

Clinical trial (NCT number)	Tumor(s) *Clinical setting*	Design	Treatment arms	Primary endpoint(s)
ANBL1531 (NCT03126916)	Neuroblastoma *First line*	Phase III Randomised *n* = 658	1: TC + CEM, tandem ASCR 2: [^131^I]mIBG, TC + CEM, tandem ASCR 3: [^131^I]mIBG, HD BuMel, single ASCR (^a^discontinued)	3-year EFS
OPTIMUM (NCT03561259)	Neuroblastoma *Progression*	Phase II *n* = 60	1: [^131^I]mIBG monotherapy 2: [^131^I]mIBG, vorinostat	Tumor response
MiNivAN (NCT02914405)	Neuroblastoma *Refractory* *Progression* *Relapse*	Phase I *n* = 36	1: [^131^I]mIBG, nivolumab 2: [^131^I]mIBG, nivolumab, low dose dinutuximab 3: [^131^I]mIBG, nivolumab, full dose dinutuximab	Safety/tolerability
NCT02378428	Neuroblastoma *Refractory* *Progression* *Relapse*	Phase II *n* = 65	Single arm: [^131^I]mIBG	Tumor response
NCT00107289	Neuroblastoma, Paraganglioma, Pheochromocytoma *Refractory* *Progression* *Relapse*	Phase II *n* = 200	Single arm: [^131^I]mIBG	Tumor response
LuDO-N (NCT04903899)	Neuroblastoma *Refractory* *Relapse*	Phase II *n* = 24	Single arm: [^177^Lu]Lu-DOTATATE	Tumor response
NEUROBLU-02 (NCT03966651)	Neuroblastoma *Refractory* *Relapse*	Phase I *n* = 18	Single arm: [^177^Lu]Lu-DOTATATE	Safety
NETTER-P (NCT04711135)	Gastroenteropancreatic neuroendocrine *Refractory* *Progression* *Relapse*	Phase II *n* = 11	Single arm: [^177^Lu]Lu-DOTATATE	Safety Radiation dose
NCT02236910	SSTR-positive	Phase II *n* = 66	Single arm: [^177^Lu]Lu-DOTATATE	Tumor response
NCT01876771	Neuroendocrine Paraganglioma Pheochromocytoma	Phase II *n* = 500	Single arm: [^177^Lu]Lu-DOTATATE	Tumor response 1-year PFS

Abbreviations: [^131^I]mIBG, *meta*-[^131^I]iodobenzylguanidine; [^177^Lu]Lu-DOTATATE, lutetium-177 labeled DOTA-Tyr3-octreotate; ASCR, autologous stem cell rescue; BuMel, busulfan melphalan; CEM, carboplatin etoposide melphalan; EFS, event-free survival; HD, high-dose; PFS, Progression free survival; TC, thiotepa cyclophosphamide.

a The third arm of the ANBL1531 trial was discontinued due to toxicity.

In other NET-expressing tumors, such as paraganglioma and pheochromocytoma (PPGL), [^131^I]mIBG has already received FDA approval for the treatment of locally advanced or metastatic disease, in both adults and pediatric patients older than 12 years old. The critical trial for this approval was a phase II single-arm study, open for adults and adolescents, achieving partial tumor response in 23% and stable disease in 69% of patients.^38^

Another established target for RLT in neural crest and neuroendocrine tumors is the somatostatin receptor (SSTR) family, particularly the subtype 2 (SSTR_2_). Early pediatric studies in neuroblastoma patients demonstrated the feasibility and safety of using [^68^Ga]Ga-DOTATATE to select children for **^177^Lu-labeled DOTATATE ([^177^Lu]Lu-DOTATATE)** therapy, based on tumor SSTR_2_ expression.^39^ In a retrospective study, in the palliative setting, peptide receptor radioligand therapy (PRRT) with [^177^Lu]Lu-DOTATATE achieved symptomatic and imaging responses in all patients.^40^ This was encouraging, as these patients had previously failed multiple lines of treatment, including [^131^I]mIBG. Thus, the prospective LuDO trial (ISRCTN98918118) was set up to evaluate the safety and activity of [^177^Lu]Lu-DOTATATE, administered in 4 cycles, at 8- to 12-week intervals, in the refractory/relapsed setting.^41^ Due to the absence of objective responses, the trial did not proceed to the second stage. However, the trial design had a few limitations, such as the long interval between cycles, which was based on adult neuroendocrine tumor experience,^8^ but suboptimal for such a rapidly proliferating tumor as high-risk neuroblastoma. Therefore, the LuDO-N phase II trial (NCT04903899), with an intensified and personalized dosing schedule, was opened and is currently recruiting patients.^42^ Concurrently, the NEUROBLU-02 phase I trial (NCT03966651) is assessing the maximum tolerated dose (MTD) of 2 cycles of [^177^Lu]Lu-DOTATATE administered at 6-week intervals.

[^177^Lu]Lu-DOTATATE has also shown antitumor activity in PPGL and neuroendocrine tumors.^43–45^ Safety and efficacy data from the pediatric phase II NETTER-P trial, combined with the adult data from the phase III NETTER-1 and NETTER-2 trials granted the approval of [^177^Lu]Lu-DOTATATE for the treatment of gastroenteropancreatic neuroendocrine tumors in children > 12 years old.^46^ The NETTER-P trial (NCT04711135) recruitment is complete, but long-term follow-up is still ongoing. Two other phase II trials are also ongoing but only patients over 14 years old are eligible for inclusion. Further clinical evidence is still required to extend the indication to other SSTR_2_-expressing tumors. To explore strategies to enhance therapeutic efficacy, the first combination trial of [^177^Lu]Lu-DOTATATE and a poly(ADP-ribose) polymerase (PARP) inhibitor is ongoing–the LuPARPed trial (NCT06607692).

Beyond [^177^Lu]Lu-DOTATATE, other SSTR_2_-targeted agents have also been investigated. **Yttrium-90 labeled DOTATOC ([^90^Y]Y-DOTATOC)** showed an encouraging disease control rate of 41% in children and young adults with refractory solid tumors, without DLTs. However, due to its physical properties (pure beta-emitter and long particle range), imaging was complex and the therapeutic window was narrow, hindering further development. Additionally, the higher incidence of renal toxicity associated with ^90^Y in adult studies led to a gradual shift toward alternative radionuclides (eg, ^177^Lu), thereby hindering studies in pediatrics as well. **[^177^Lu]Lu-DOTATOC** is under investigation in the ongoing phase I KinLET trial (NCT06441331) (Table 2), and **copper-67-labeled SARTATE ([^67^Cu]Cu-SARTATE)**, developed as the therapeutic counterpart to copper-64-labeled SARTATE ([^64^Cu]Cu-SARTATE), was scheduled for evaluation in a phase I/II study in children and adolescents with high-risk neuroblastoma (NCT04023331). However, this study was discontinued following a sponsor decision, and no further development is currently planned.

**Table 2:** Ongoing radioligand therapy trials in pediatric patients (mixed cohorts).

Clinical trial (NCT number)	Tumor(s) *Clinical setting*	Design	Treatment arms	Primary endpoint(s)
LuPARPed (NCT06607692)	SSTR-expressing *Refractory* *Relapse*	Phase II *n* = 25	Single arm: [^177^Lu]Lu-DOTATATE, Olaparib	ORR
KinLET (NCT06441331)	SSTR-expressing	Phase I *n* = 20	Single arm: [^177^Lu]Lu-DOTATOC	pediatric dose

Abbreviations: [^177^Lu]Lu-DOTATATE, lutetium-177 labeled DOTA-Tyr3-octreotate; [^177^Lu]Lu-DOTATOC, lutetium-177 labeled DOTA-Tyr3-octerotide; ORR, overall response rate; SSTR, somatostatin receptor.

### Future perspectives

Within the NET-targeting domain, [^131^I]mIBG therapy continues to demonstrate modest overall response rates, highlighting a critical need to identify responders more effectively. Theranostic pairing with diagnostic tracers such as [^124^I]mIBG or [^18^F]mFBG may enable more precise responder selection and facilitate individualized dosimetry.^47–49^ In parallel, combination regimens (eg, with chemotherapy or radiosensitisers) have already shown improved response rates and may ultimately translate into improved event-free and overall survival, particularly if guided by theranostics. However, continued development of [^131^I]mIBG therapy seems threatened by flaws in global supply logistics, which have already disrupted clinical trials (ie, SIOPEN phase III HR-NBL2 study-arm VERITAS, and COG phase III study ANBL 1531).

A different approach to SSTR_2_ targeting is through receptor antagonists (eg, OPS120), which do not rely on receptor internalization and can bind to a greater number of receptor conformations, resulting in higher overall tumor uptake.^50–53^ If applied to RLT, these SSTR_2_ antagonists could overcome resistance to current SSTR2 agonist-based therapies, and expand eligibility to patients with borderline or moderate SSTR_2_ expression.

Emerging alpha-emitters including astatine-221 (^221^At), lead-212 (^212^Pb), and actinium-225 (^225^Ac) have the potential to offer higher tumor-cell killing capacity and reduced off-target toxicity compared to beta-emitters, due to their higher linear energy transfer and shorter tissue range, warranting further investigation (Figure 3).^54–57^ Alternative radiopharmaceuticals using terbium-161 (^161^Tb) and copper-67 (^67^Cu) may also offer improved tumor killing capacity compared to ^177^Lu, although toxicity, response, and survival data remain to be established in pediatric populations.

## Sarcomas

Pediatric sarcomas are a heterogeneous group of mesenchymal malignancies accounting for approximately 10%-15% of all childhood cancers.^58^ These tumors are broadly divided into 2 categories: bone sarcomas, primarily osteosarcoma and Ewing sarcoma; and soft tissue sarcomas (STS), with rhabdomyosarcoma (RMS) being the most prevalent subtype.^58^

The main strategy for targeting bone sarcomas, such as osteosarcoma, is to take taking advantage of the uncontrolled osteoblastic activity, which requires high amounts of phosphonates and calcium. This need is exploited by attaching radionuclides to a phosphonate molecule or to radionuclides that mimic calcium’s chemical properties. **Samarium-153 (^153^Sm) labeled EDTMP ([^153^Sm]EDTMP)**, a phosphonate-containing radiopharmaceutical that binds to hydroxyapatite at sites of high bone turnover, is usually used for palliative treatment of bone metastases in adults but has also been used in children with osteosarcoma (Figure 4). Although very high tumor doses were achieved (240 Gy) and symptoms improved,^59^ sustained response was rarely observed, even in combination with chemotherapy.^60^ Increased-dose protocols with autologous stem cell support achieved disease stabilization, but not tumor response.^61^^,^^62^ An ongoing phase I trial (NCT06008483) is evaluating a similar, potentially more stable and less toxic radiopharmaceutical, **[^153^Sm]DOTMP**, in patients over 15 years old with bone tumors or bone metastasis (Table 3). Another example is **rhenium-186 (^186^Re) labeled HEDP ([^186^Re]HEDP)**, which was administered in combination with external beam radiation therapy (EBRT) to 13 patients with inoperable osteosarcoma.^63^ The combination was not sufficient to achieve tumor sterilization, and further development was not pursued. However, **radium-223 dichloride ([^223^Ra]RaCl_2_)**, an alpha-emitter earth alkali-metal that mimics calcium, recently showed encouraging safety and antitumor activity (metabolic response of primary and metastatic lesions) in children with recurrent or metastatic osteosarcoma.^64^ Surprisingly, children tolerated twice the amount of activity compared to adults, which clearly illustrates the value of pediatric phase I data.

**Figure 4:**
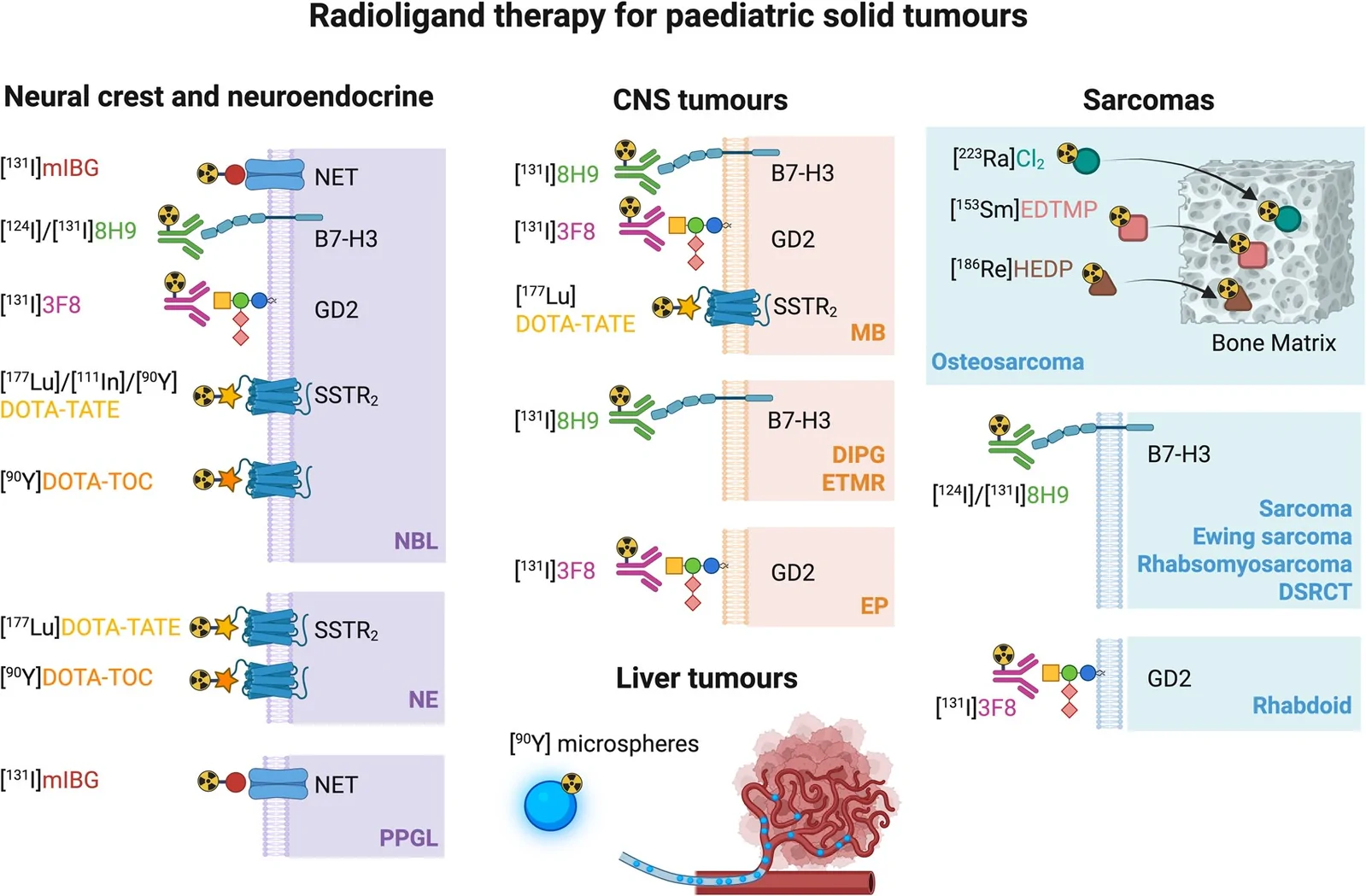
Overview of radioligand therapy (RLT) in pediatric solid tumors. Abbreviations: ^90^Y = yttrium-90; ^111^In = indium-111; ^124^I = iodine-124; ^131^I = iodine-131; ^153^Sm = samarium-153; ^177^Lu = lutetium-177; ^186^Re = rhenium-186; ^223^Ra = radium-223; 3F8 = anti-GD2 monoclonal antibody; 8H9 = Omburtamab; DIPG = diffuse intrinsic pontine glioma; DSRCT = desmoplastic small round cell tumor; EP = ependymoma; ETMR = embryonal tumor with multilayered rosettes; GD2 = disialoganglioside-2; MB = medulloblastoma; NBL = neuroblastoma; NE = neuroendocrine; NET = norepinephrine transporter; PPGL = pheochromocytoma and paraganglioma; SSTR_2_ = somatostatin receptor subtype 2.

**Table 3:** Ongoing radioligand therapy trials in pediatric patients with sarcomas.

Clinical trial (NCT number)	Tumor(s) *Clinical setting*	Design	Treatment arms	Primary endpoint(s)
NCT06008483	Bone tumors, bone metastasis	Phase I *n* = 17	Single arm: [^153^Sm]DOTMP	DLT rate
NCT04022213	Desmoplastic small round cell tumor, other tumors of the peritoneum	Phase II *n* = 31	Single arm: [^131^I]I-Omburtamab	PFS

Abbreviations: [^153^Sm]DOTMP; samarium-153 labeled 1,4,7,10-tetraazacyclododecane-1,4,7,10-tetramethylenephosphonic acid; [^131^I]I-Omburtamab, iodine-131 labeled Omburtamab; DLT, dose limiting toxicity; PFS, progression-free survival.

Experience is more limited in pediatric soft tissue sarcomas (STS), but tumor-associated surface antigens hold promise for RLT. One such antigen is B7-H3, an immune-regulatory protein overexpressed in several pediatric solid tumors.^65^ In a phase I clinical trial, patients with desmoplastic small round cell tumor (DSCRT), a type of STS associated with poor prognosis, were treated with the intraperitoneal radiolabeled monoclonal antibody Omburtamab ([^131^I]I-Omburtamab).^66^ Intraperitoneal [^131^I]I-Omburtamab was safe and showed a favorable biodistribution profile. Although most patients had DSRCT, a small number of rhabdomyosarcoma (RMS) and Ewing sarcoma cases were included, highlighting the potential of B7-H3-targeted RLT for other types of pediatric STS. A phase II study, combining [^131^I]I-Omburtamab and external beam radiotherapy is ongoing, aiming to improve survival (Table 3).

### Future perspectives

Heterogeneous target expression remains a major challenge for RLT in pediatric sarcomas. While bone-seeking agents can overcome this in osteoblastic lesions, only [^153^Sm]EDTMP has established pediatric dose-optimization data. Initial findings with [^223^Ra]RaCl_2_ show a large safety margin, but phase II data and integration with the diagnostic tracer [^18^F]NaF for patient selection are still needed. The long-term efficacy of [^223^Ra]RaCl_2_ will likely be limited by populations of cells capable of escaping the osteoblastic phenotype,^67^ so future prospective studies will need to focus on combination therapy with other agents.

Disialoganglioside-2 (GD2) is a promising target expressed by osteosarcoma, Ewing sarcoma, RMS, and other STS subtypes.^68^^,^^69^ Additionally, fibroblast activation protein (FAP) and prostate-specific membrane antigen (PSMA) have been successful targets in adult RLT and show potential for translation to pediatric oncology.^70^ Nevertheless, all these targets require further validation and development.

Overall, pediatric sarcomas remain underrepresented in RLT trials compared with other pediatric solid tumors, highlighting the urgent need for multicenter clinical studies that integrate biologically relevant targets, theranostic imaging, pediatric dose-optimization, and combination strategies.

## CNS tumors and leptomeningeal metastases

CNS tumors are the most common solid malignancies in children and a leading cause of pediatric cancer mortality.^71^ Incidence shifts by age from embryonal tumors (eg, medulloblastoma) in younger children to glial tumors (eg, astrocytoma) in adolescents. A main hurdle to RLT efficacy in CNS tumors and leptomeningeal metastases (LM) is the blood-brain barrier/blood-tumor barrier (BBB/BTB), which severely restricts drug delivery.^72^

Strategies to bypass the BBB/BTB or facilitate drug delivery include local administration (ie, intraventricular administration), convection-enhanced delivery (CED), focused ultrasound (FUS), or innovatively designed drugs.^72^ Most evidence focuses on local delivery of B7-H3-targeting **[^131^I]I-Omburtamab**^73–78^ and **[^124^I]I-Omburtamab**.^79^^,^^80^ Notably, a phase I dose-escalation trial used [^124^I]I-Omburtamab as a theranostic agent in patients with diffuse intrinsic pontine glioma (DIPG), setting the maximum tolerated dose (MTD) at 222 MBq via CED.^80^ Its theranostic properties enabled real-time kinetic monitoring and image-based dosimetry. [^131^I]I-Omburtamab was used to treat patients with medulloblastoma and ependymoma in a long-term follow-up study,^78^ showing median OS of 1.9 and 6.7 years, respectively, and a phase II trial (NCT04743661) is ongoing (Table 4).

**Table 4:** Ongoing radioligand therapy trials in pediatric patients with brain tumors and leptomeningeal metastases.

Clinical trial (NCT number)	Tumor(s) *Clinical setting*	Design	Treatment arms	Primary endpoint(s)
NCT04743661	Medulloblastoma Ependymoma *Refractory* *Progression* *Relapse*	Phase II *n* = 62	Single arm: [^131^I]I-Omburtamab	2-year EFS
NCT05278208	High grade CNS tumors, Meningioma	Phase I/II *n* = 65	Single arm: [^177^Lu]Lu-DOTATATE	MTD, RP2D, Safety, PFS
NCT07061626	Ependymoma, High-grade glioma *Refractory* *Progression* *Recurrent*	Phase I *n* = 56	Single arm: [^186^Re]Re-nanoliposome	MTD, ORR, 1-year PFS

Abbreviations: [^131^I]I-Omburtamab, iodine-131 labeled Omburtamab; [^177^Lu]Lu-DOTATATE, lutetium-177 labeled DOTA-Tyr3-octreotate; [^186^Re]Re-nanoliposome, rhenium-186 labeled nanoliposome; CNS, central nervous system; EFS, event-free survival; MTD, maximum tolerated dose; ORR, objective response rate; PFS, progression-free survival; RP2D, recommended phase II dose.

The GD2-targeting radiolabeled mAb **[^131^I]I-3F8** has been mostly used to treat patients with LM from GD2-expressing tumors.^73^^,^^81–83^ but has also shown improved survival (OS) rates (5-year OS = 45%) in patients with high-risk and recurrent medulloblastoma,^83^ compared with historical data (3-year OS = 14%).^84^

Another target for RLT in pediatric brain tumors, especially medulloblastoma, is the SSTR family. Use of **[^177^Lu]Lu-DOTATATE** is being explored in a phase I/II trial, in patients with recurrent or progressive high-grade CNS tumors and meningiomas. In parallel, another phase I/IIa trial will explore a novel approach using rhenium-186 nanoliposomes **([^186^Re]Re Nanoliposome)—**microscopic lipid-bilayer vesicles that encapsulate the radioactive payload—delivered via CED directly into the tumor, in children with recurrent, refractory, or progressive ependymoma and high-grade glioma.

### Future perspectives

Although B7-H3 and GD2 are promising targets for RLT in CNS tumors, further work is required to better characterize their expression, define their relationship with response, and understand mechanisms of resistance. Given the heterogeneity of certain CNS tumors, such as DIPG, identifying and validating additional tumor-specific targets is a clear need.

In adult neuro-oncology, [^131^I]Iodofalan is emerging as the therapeutic counterpart to amino acid-based positron emission tomography (PET) imaging. It targets the L-type amino acid transporter 1 (LAT1), a protein overexpressed in tumor cells that allows BBB/BTB bypassing via active transport. Following safety and dosimetry data from the IPAX-1 trial,^85^ [^131^I]Iodofalan has now progressed to the IPAX BrIGHT phase III trial (NCT07100730). Should the results establish efficacy, a clear pathway for pediatric translation emerges, as many pediatric CNS tumors demonstrate increased uptake on amino acid PET imaging.^86^^,^^87^ Parallel development of radiopharmaceuticals incorporating other radionuclides (eg, [^177^Lu]Lu-Omburtamab) and capable of crossing the BBB may further enhance the therapeutic armamentarium.^80^

Local delivery of RLT is also a promising strategy to overcome the BBB/BTB, as it enables a higher tumor dose while reducing systemic toxicity. However, broader implementation will require technique and protocol standardization as well as radiation safety assurance. Theranostic approaches can further aid development through optimal selection of patients, and by enabling dosimetry and (visual) confirmation of drug retention.

Given the vulnerability of the developing brain, systematic long-term evaluation of neurocognitive sequelae should also be incorporated into future trials, particularly for lower-grade tumors. Finally, larger multicenter studies are warranted to confirm survival benefits and to define optimal combinations of RLT with existing modalities such as surgery, radiotherapy, and systemic therapies.

## Hepatic tumors

Childhood liver tumors are rare, accounting for around 1%-2% of pediatric malignancies. While hepatoblastoma is the predominant subtype, typically occurring in children younger than 3 years old, pediatric hepatocellular carcinoma (HCC) is more frequent in older children and adolescents.^88^ Outcomes have markedly improved, but prognosis remains poor for patients with unresectable, metastatic, or chemotherapy-refractory disease. For these patients, locoregional approaches, such as transarterial radioembolization (TARE) with **^90^Y-labeled microspheres** have been investigated in small series and case reports.^89–94^ A prospective trial evaluating the clinical outcomes and quality of life after TARE in children, adolescents, and young adults with hepatic tumors was set up (NCT04315883) but has been discontinued due to sponsor decision.

### Future perspectives

Current evidence regarding pediatric TARE is promising but larger studies are required to define safety, efficacy, and long-term outcomes, and to develop standardization on dosimetry, delivery techniques, and patient selection criteria. In adults with HCC, TARE has increasingly been used prior to surgical intervention as a bridge to transplantation, for downstaging, and as a means of assessing tumor behavior and aggressiveness. Translating this to children will be challenging, not only due to the small patient population, but also because TARE requires a trained team of nuclear medicine physicians and (pediatric) interventional radiologists. Nevertheless, future research is warranted given the promising results in pediatric case series and the established efficacy of TARE in adult populations.

## Overarching considerations

Pediatric cancers are biologically distinct from adult malignancies, limiting the direct translation of RLTs developed in adult oncology. This challenge is compounded by the rarity of pediatric solid tumors, which reduces the financial incentive for developing pediatric-specific RLTs. In parallel, adult-derived protocols are not always directly applicable, as seen in the transition between the NETTER-1 and LuDO-N trials. Consequently, a key question emerges: should pediatric RLT focus on translating established adult compounds, or on adopting flexible trial designs that allow early integration of novel agents? Regardless of the answer, dedicated pediatric phase I studies are essential to define safe and effective dosing, pharmacokinetics, and organ-specific toxicity thresholds, and to reduce the dependency on extrapolated data from adult studies and small case series.

Dosing strategies remain a central issue in pediatric RLT. Fixed-activity regimens are simpler to implement across centers, but fail to account for patient heterogeneity, an especially important consideration in pediatric oncology, where organ size and proportions vary substantially from infancy to adolescence. The same administered activity results in markedly different tumor and organ doses. Weight-based dosing only partially addresses this issue, as patients with similar body weight may still require different activities, depending on tumor burden, biodistribution, and target expression. Conversely, dosimetry-guided approaches enable individualized treatment planning, maximizing tumor dose while limiting exposure to critical organs, and allow adaptive strategies, such as dose escalation in low-uptake tumors or fractionation based on early pharmacokinetics. However, dosimetric regimens are resource-intensive, requiring serial imaging, specialized software, and dedicated expertise. A hybrid approach may therefore be the most sensible, using fixed or weight-based dosing in early phases, before transitioning to dosimetry-guided optimization as experience matures.

Safety remains a major concern given children’s vulnerability to both acute and long-term toxicities. Hematologic toxicity is the primary adverse effect across most RLTs and has been most extensively characterized for [^131^I]mIBG in neuroblastoma. For renally excreted agents, such as small molecules, renal toxicity must be actively prevented by careful monitoring and protective strategies. Interestingly, emerging data from alpha-emitters (ie, ^223^RaRCl_2_) challenges the assumption that children invariable tolerate lower administered activities. Long-term safety remains incompletely defined, even for well-established RLTs such as [^131^I]mIBG. Late effects including thyroid damage, hypothyroidism, and secondary thyroid malignancies have been reported despite appropriate protection at the time of treatment, and gonadal toxicity has also been described. This demonstrates the need for systematic, long-term follow-up in pediatric patients receiving RLT, for both novel and established compounds.

Practical and logistical challenges further complicate implementation. Current radioprotection protocols often require post-therapy isolation, imposing considerable emotional strain on patients and families. Here, alpha emitters may offer an advantage, as their short path length and minimal external radiation exposure eliminate the need for isolation. Furthermore, pediatric RLT requires specialized infrastructure, trained personnel, and complex workflows, particularly when multi-time-point imaging with sedation or anesthesia is needed for dosimetry or theranostic approaches. Finally, global radionuclide supply instabilities remain a critical barrier to equitable access, as demonstrated by the disruptions to the VERITAS and ANBL1531 trials.

Addressing these hurdles will require pediatric-specific translational research, innovative trial designs, and close collaboration among academia, industry, and regulatory bodies. Ultimately, the successful translation of RLT into pediatric oncology depends not only on new targets, improved radiochemistry, and technological advances, but also on the development of clinical frameworks tailored to the unique biological, developmental, and societal context of treating children with solid tumors.

## Supplementary Material

tqag161_Supplementary_Data
