# Supplementary material for: Radioligand therapy for pediatric patients with solid tumors
Source: Br J Radiol. 2026 Jul 7;99(1186):2080–92. doi: 10.1093/bjr/tqag161 (PMC13623218; doi:10.1093/bjr/tqag161)
Supplement: Supplementary file 1 [file tqag161_supplementary_data.zip › Supplemental Data.docx]

**SUPPLEMENTAL DATA – Search Strings**

**PubMed search strategy**

Search performed on 10/12/2025

Filters:

Language: English

Publication date: 2000 - present

Concept A – Radioligand / Radiopharmaceutical therapy

("Radiopharmaceuticals"[Mesh] OR radioligand therap*[tiab] OR targeted radionuclide therap*[tiab] OR therapeutic radiopharmaceutical*[tiab] OR molecular radiotherap*[tiab] OR radioimmunotherap*[tiab] OR "Peptide Receptor Radionuclide Therapy"[tiab] OR PRRT[tiab] OR 131I-MIBG[tiab] OR 177Lu-DOTATATE[tiab] OR lutetium-177[tiab] OR yttrium-90[tiab] OR actinium-225[tiab] OR Omburtamab[tiab] OR iodine-125[tiab] OR radium-223[tiab] OR radium[tiab] OR alpha particle*[tiab] OR astatine[tiab])

Concept B – Paediatric population

("Child"[Mesh] OR "Infant"[Mesh] OR "Adolescent"[Mesh] OR "Pediatrics"[Mesh] OR pediatric*[tiab] OR paediatric*[tiab] OR child*[tiab] OR adolescen*[tiab])

Concept C – Solid tumours

("Neoplasms"[Mesh] OR solid tumor*[tiab] OR solid tumour*[tiab] OR neuroblastoma[tiab] OR sarcoma*[tiab] OR neuroendocrine tumor*[tiab] OR osteosarcoma*[tiab] OR pheochromocytoma[tiab] OR paraganglioma[tiab] OR Ewing[tiab])

Concept D – Study type

("Clinical Trial"[pt] OR "Clinical Trial, Phase I"[pt] OR "Clinical Trial, Phase II"[pt] OR "Clinical Trial, Phase III"[pt] OR "Case Reports"[pt] OR case report*[tiab] OR case series[tiab] OR retrospective stud*[tiab] OR prospective stud*[tiab])

Concept E – Exclusion of reviews

(18F-FDG[tiab] OR FDG[tiab] OR fluorodeoxyglucose[tiab] OR 123I-MIBG[tiab] OR

iodine-123-MIBG[tiab] OR iobenguane I-123[tiab])

Concept F – Exclusion of diagnostic radiotracers

(18F-FDG[tiab] OR FDG[tiab] OR fluorodeoxyglucose[tiab] OR123I-MIBG[tiab] OR

iodine-123-MIBG[tiab] OR iobenguane I-123[tiab])

Final combined PubMed search query

(A) AND (B) AND (C) AND (D) NOT (E) NOT (F)

**Embase search strategy**

Search performed on 12/12/2025

Concept 1 – Radiopharmaceutical / Radionuclide Therapy

'radiopharmaceutical'/exp OR 'radionuclide therapy'/exp OR 'radioimmunotherapy'/exp OR 'radioligand therapy'/exp OR radioligand therap*:ti,ab OR targeted radionuclide therap*:ti,ab OR therapeutic radiopharmaceutical*:ti,ab OR molecular radiotherap*:ti,ab OR radioimmunotherap*:ti,ab OR 'peptide receptor radionuclide therapy':ti,ab OR prrt:ti,ab OR '131i mibg':ti,ab OR 'iobenguane':ti,ab OR '177lu dotatate':ti,ab OR lutetium-177:ti,ab OR yttrium-90:ti,ab OR actinium-225:ti,ab OR astatine-211:ti,ab OR omburtamab:ti,ab OR iodine-125:ti,ab OR radium-223:ti,ab OR alpha particle*:ti,ab

Concept 2 – Paediatric population

'infant'/exp OR 'child'/exp OR 'adolescent'/exp OR 'pediatrics'/exp OR pediatric*:ti,ab OR

paediatric*:ti,ab OR child*:ti,ab OR adolescen*:ti,ab

Concept 3 – Solid tumours

'neoplasm'/exp OR 'solid tumor':ti,ab OR 'solid tumour':ti,ab OR neuroblastoma:ti,ab OR sarcoma*:ti,ab OR osteosarcoma*:ti,ab OR ewing*:ti,ab OR neuroendocrine tumor*:ti,ab OR pheochromocytoma:ti,ab OR paraganglioma:ti,ab

Study Type Filter

'clinical study'/de OR 'clinical trial'/de OR 'phase 1 clinical trial'/de OR 'phase 2 clinical trial'/de OR 'phase 3 clinical trial'/de OR 'case report'/de OR case report*:ti,ab OR case series:ti,ab OR retrospective stud*:ti,ab OR prospective stud*:ti,ab

Limits

[humans]/lim AND [english]/lim AND [2000-2026]/py

Final Query

(Concept 1) AND (Concept 2) AND (Concept 3) AND (Study Type Filter) AND [humans]/lim AND [english]/lim AND [2000-2026]/py
